# Layer segmented filamentous bacteria colonize and impact gut health of broiler chickens

**DOI:** 10.1128/msphere.00492-24

**Published:** 2024-10-18

**Authors:** Jared Meinen-Jochum, Caleb J. Skow, Melha Mellata

**Affiliations:** 1 Interdepartmental Microbiology Graduate Program, Iowa State University, Ames, Iowa, USA; 2 Department of Food Science and Human Nutrition, Iowa State University, Ames, Iowa, USA; University of Wyoming College of Agriculture Life Sciences and Natural Resources, Laramie, Wyoming, USA

**Keywords:** segmented filamentous bacteria, *Enterobacteriaceae*, *Campylobacter jejuni*, intestinal barrier function, cytokines, broiler chickens

## Abstract

In commercial poultry farms, chicks hatch away from their progenitors from which they acquire key host-specific microbiota, like segmented filamentous bacteria (SFB) involved in gut maturation in early life. This study investigated whether providing chicken SFB to newly hatched broilers would increase their gut maturation and resistance to bacteria relevant to broiler and human health. One-day-old Ross308 broilers were orally treated with either phosphate-buffered saline (CON) or layer-derived SFB (D-SFB). On days 5, 10, 17, and 24, feces were collected to detect and enumerate SFB and *Enterobacteriaceae*. On days 8, 15, 22, and 29, birds were euthanized, intestinal samples were collected to detect and enumerate SFB through quantitative PCR (qPCR) and microscopy and expression of genes associated with gut immune function through reverse transcription-qPCR. This study showed that, despite their host specificity, layer SFB can colonize their genetically distinct relative broilers. Ileal SFB colonization was accelerated by a week with the SFB treatment and covered the proximal, medial, and distal sections of the ileum. Colonization of the ileum by SFB in early life highly activated gene expression of intestinal barrier proteins and cytokines, e.g., IL-10 and IFNγ but not IL-17. SFB treatment reduced the level of *Enterobacteriaceae* in the gut and provided superior resistance to intestinal and extraintestinal pathogens as tested *in vitro*. Overall, early gut colonization of SFB is imperative for the maturation of the gut immune system and the establishment of a homeostatic gut environment. Improving our understanding of gut immune maturation in food-producing animals is crucial for both human and animal health.

**IMPORTANCE:**

In commercial farms, newly hatched chicks may lack host-specific microbiota that help mature their gut immune system for lifelong health benefits. Here, introducing an avian segmented filamentous bacteria (SFB) to commercially sourced chickens orally at hatch accelerated SFB colonization of the ileum. Remarkably, SFB from layers were able to colonize broilers and enhance gut immune maturation, and this immunomodulation impacted the ability to increase intestinal and extraintestinal resistance to bacteria relevant to poultry and human health. With the antibiotic restrictions in animal production, strategies that will help mitigate infections are urgently needed. In summary, we developed a live prophylactic for newly hatched chicks to improve animal health and food safety. Due to the host specificity of SFB, our data highlight the importance of investigating the molecular mechanism of SFB interaction in their own host.

## INTRODUCTION

Unique gut bacteria isolated from healthy animals have been brought to the forefront of gut immunology research because of their probiotic benefits to the host organism. Through interactions with the gut microenvironment, these bacteria provide benefits through alterations of the gut pH, production of secondary metabolites, and competitive exclusion of pathogenic organisms (1–3). As the field of probiotics expands, bacteria impacting the maturation of the gut immune system are increasingly attractive due to their ability to provide lifelong benefits to the host. Of these, segmented filamentous bacteria (SFB), specifically *Candidatus Arthromitus*, are gut-associated bacteria regularly found in numerous vertebrate species, including humans, pigs, mice, rats, turkeys, and recently in chickens supported by concordant observations by our team (4–7).

SFB are Gram-positive spore-forming bacteria that are closely related to Clostridia species. Through a unique symbiotic relationship with the host, SFB trigger the maturation of the gut immune system in early life (8–10). However, SFB are host-specific and will not form an intimate connection to the epithelium or even colonize genetically diverse hosts (11, 12). Furthermore, the inability to culture SFB *in vitro* has greatly inundated translational studies of SFB sourced from different organisms (13).

The early colonizing gut microbiota is even more crucial in food animals due to its link to animal health, productivity, and food safety (14–16). In commercial poultry, chicks are hatched away from their progenitors and other adult birds, from which they could inherit key host-specific microbiota. Thus, host-specific bacteria-based treatments that could drive the maturation of the gut immune system in early life are needed. Our team previously studied SFB in layers (5, 6); still, studies on SFB in broilers are warranted due to distinct genetics of layers and broilers selected for either improved feed conversion and rapid growth (broilers) or production of eggs (layers) (17, 18). Owing to the varying host-microbiota interactions between broilers and layers (19–21), understanding the role of SFB colonization and cross-colonization in both layers and broilers is necessary. Therefore, the objectives of this study were to test the ability of layer SFB to (i) effectively colonize broiler chickens; (ii) trigger gut immune maturation through the expression of immunomodulatory genes, e.g., antimicrobial peptides (AMPs), tight junction proteins, and cytokines; and (iii) provide broad intestinal and extraintestinal antimicrobial activities against bacteria relevant to broiler poultry. Understanding SFB-host interaction mechanisms in different genetic lines and developing a single treatment that can be used in layers and broilers will be cost-effective and benefit the poultry industry.

## RESULTS

### Broiler treatment with D-SFB resulted in early colonization of SFB

**Fig 1:**
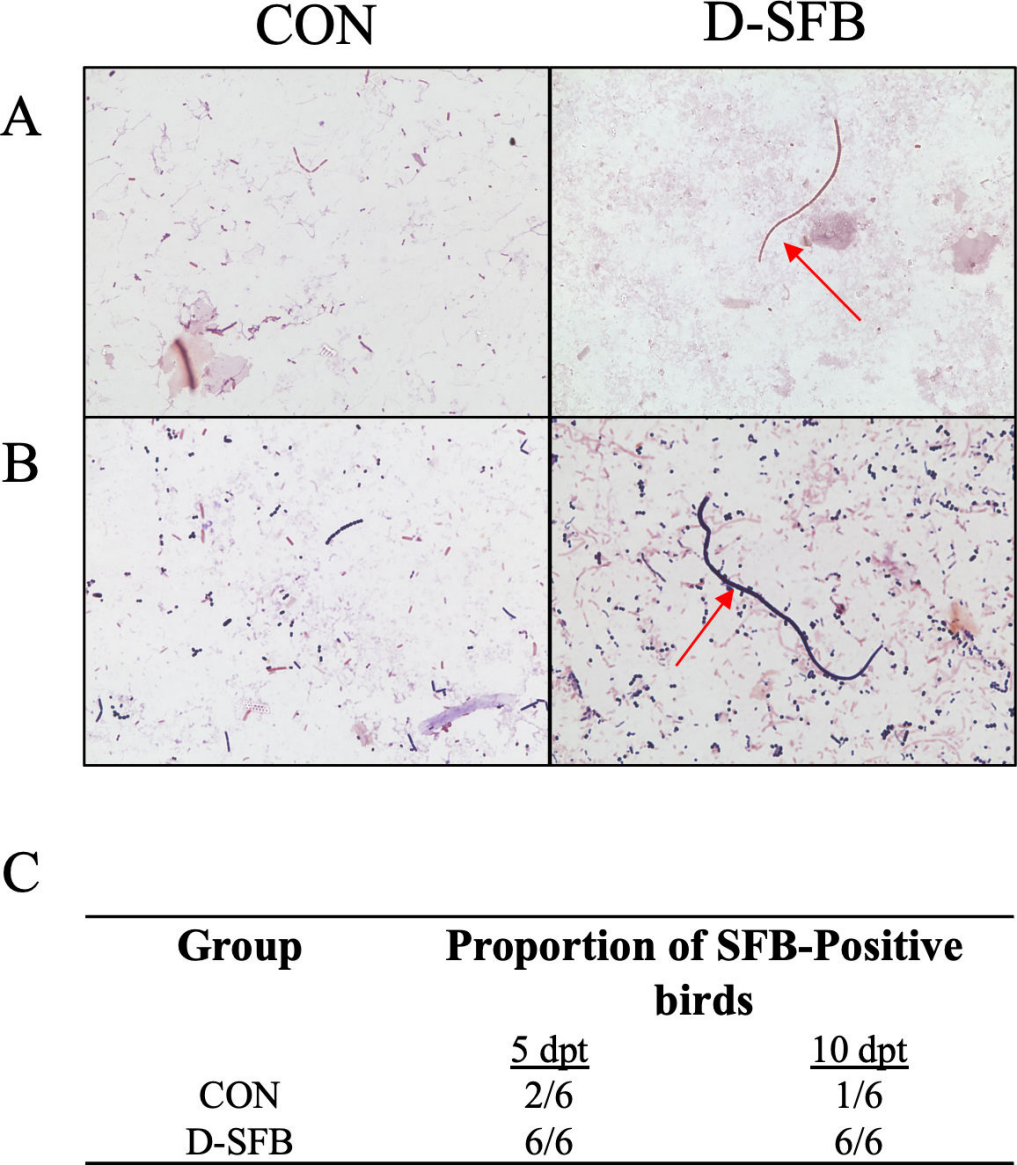
Representative images of Gram stain microscopy of feces. Feces from the control (CON) and SFB-treated birds (D-SFB) were screened via Gram stain and microscopy at (**A**) 5 and (**B**) 10 days post-treatment (dpt). (**C**) Proportion of birds that tested positive for SFB via PCR. Images were collected at 100× magnification. Red arrows indicate SFB-like bacteria.

**Fig 2:**
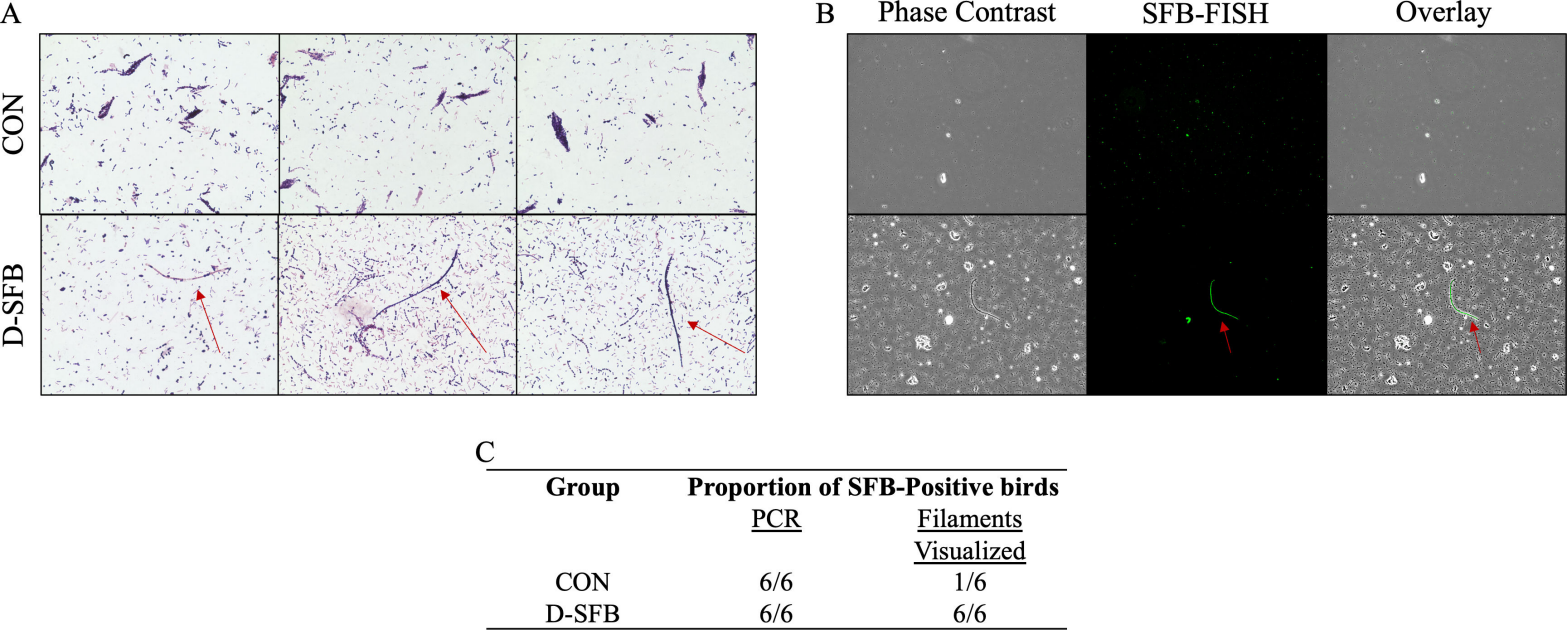
Representative Gram stain images of cecal content. Ninety random fields of vision were investigated in cecal control (CON) and SFB-treated (D-SFB) groups at 8 days post-treatment for SFB-like morphologies via Gram stain (**A**) and fluorescent *in situ* hybridation (FISH) (**B**). (**C**) Proportion of birds that tested positive for SFB via PCR at 8 dpt. All Gram stain microscopy images were taken at 100× magnification and FISH images were taken at 40× to employ phase contrast overlay. Red arrows indicate SFB-like bacteria.

PCR screening of feces, cecal content, and ileum for the presence of SFB showed that, at 5 days post-treatment (dpt), 100% (6/6) of D-SFB broilers and 33% (2/6) of CON broilers were tested positive for SFB in feces (Fig. 1). However, the Gram stain microscopy detected the distinct long segmented filamentous morphology of SFB only in D-SFB birds at 5 and 10 dpt (Fig. 1A and B). In CON birds, 17% (1/6) had only short segmented filamentous morphology of SFB, while 83% (5/6) of CON birds did not have any of these segmented filamentous morphologies of SFB (Fig. 1A and B). In the cecal content, at 8 dpt, all birds in both groups were SFB-positive via PCR. However, segmented filamentous morphologies of SFB again were only detected in D-SFB birds by Gram stain microscopy and confirmed via fluorescent *in situ* hybridization (FISH) staining (Fig. 2B and C). As both groups demonstrated colonization of the entire ileum at 15 dpt, continued examination of the ceca for SFB was not performed.

**Fig 3:**
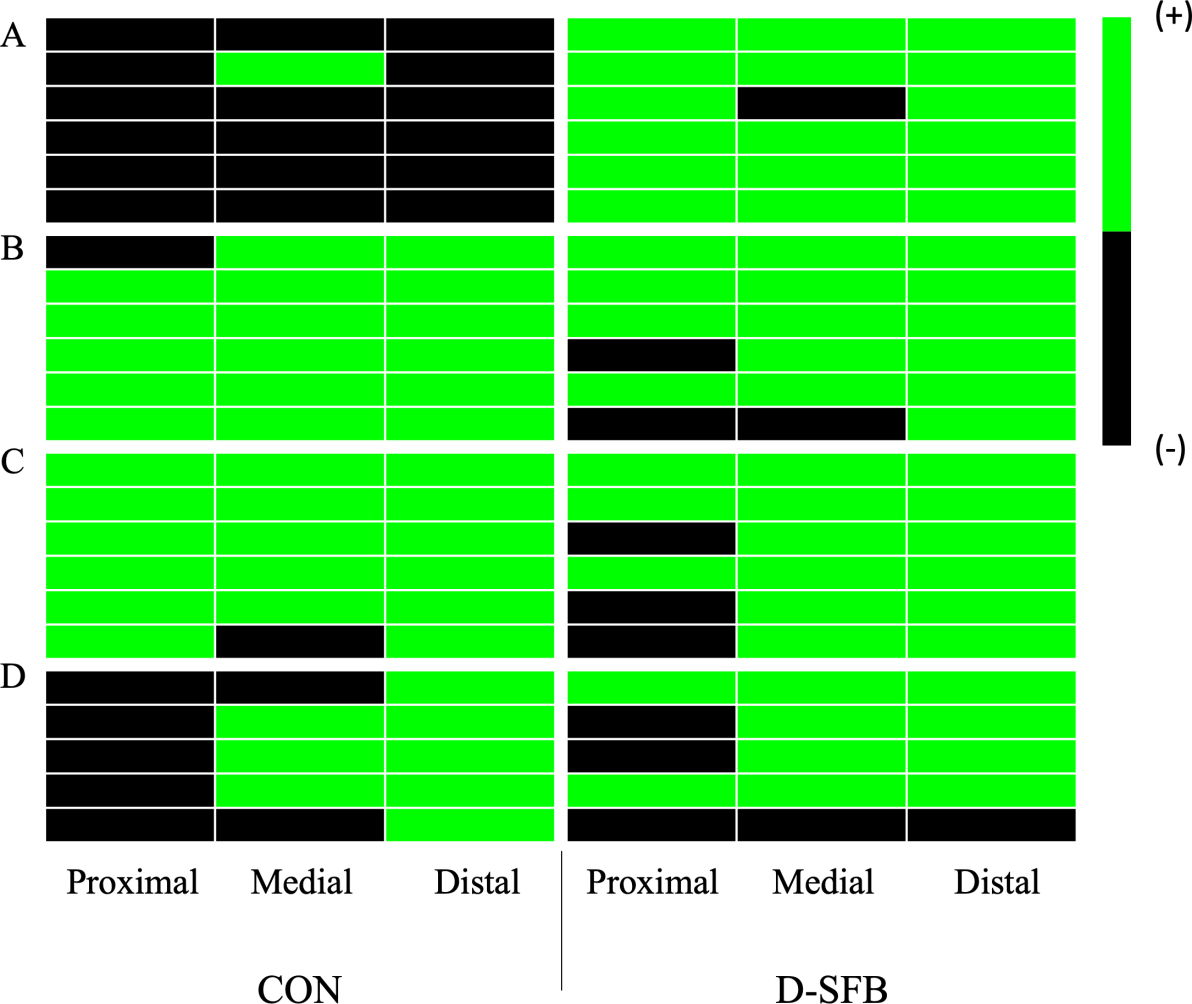
SFB Colonization throughout the broiler Ileum. Segmented filamentous bacteria (SFB) were detected in proximal, medial, and distal ileum at (**A**) 8, (**B**) 15, (**C**) 22, and (**D**) 29 days post-treatment (dpt). Each row represents an individual bird in different groups. Each box represents a separate PCR result.

**Fig 4:**
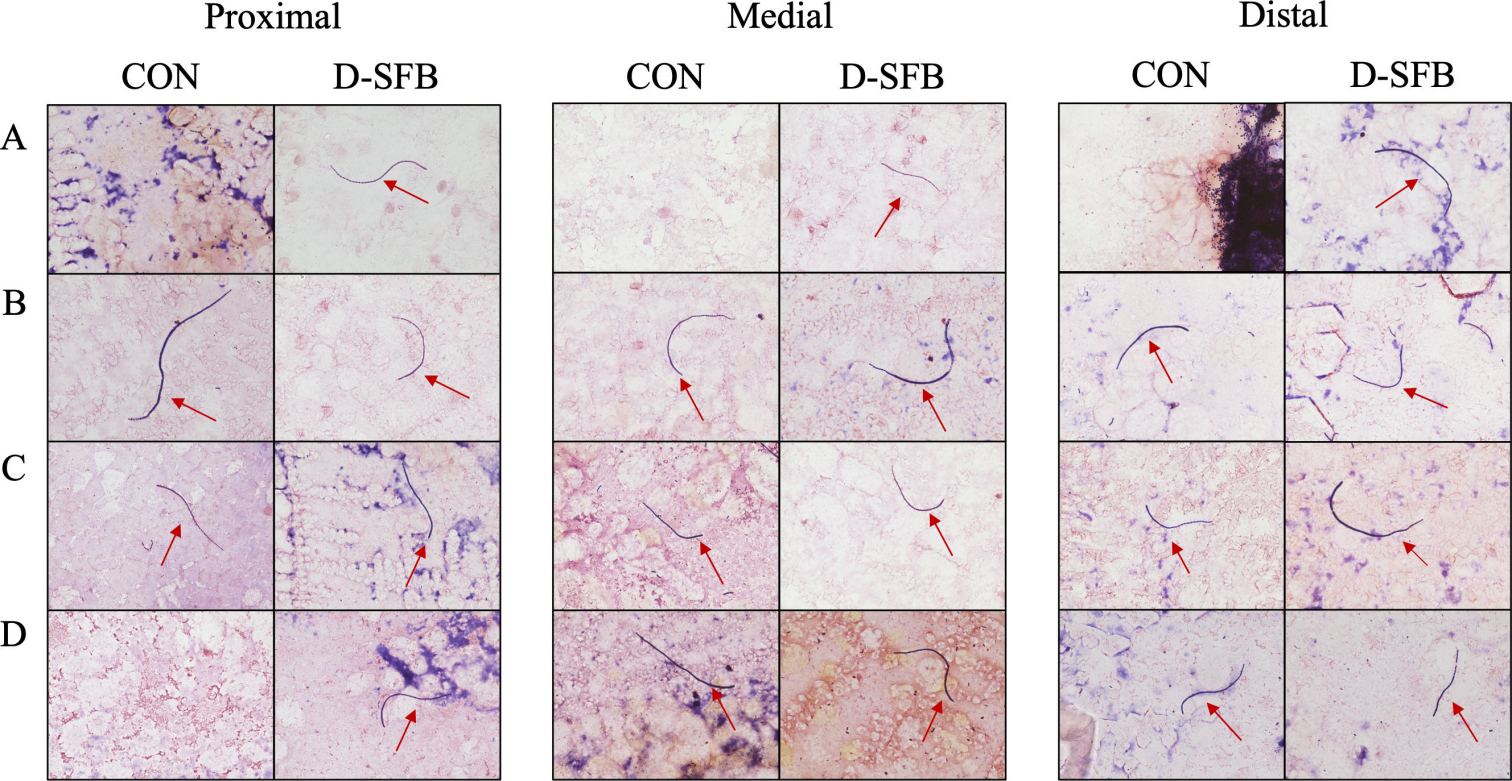
Representative images of Gram stain microscopy of broiler ilea. Proximal, medial, and distal ileum scrapings from control (CON) and SFB-treated (D-SFB) broilers at (**A**) 8, (**B**) 15, (**C**) 22, and (**D**) 29 days post-treatment (dpt). Red arrows indicate SFB-like bacteria.

In the ileum, PCR determined SFB localization throughout the tissue and further verified with Gram stain microscopy to identify distinctive SFB morphologies. At 8 dpt, only 17% (1/6) of CON birds had the medial ileum section colonized by SFB (Fig. 3A) compared to 83% (5/6) of D-SFB birds that had their proximal, medial, and distal ileum colonized with SFB and 17% (1/6) of D-SFB birds that had the proximal and distal but not the medial ileum colonized (Fig. 3A). At 15 dpt, both the CON and D-SFB birds were colonized by SFB in all three sections of the ileum tested (Fig. 3B). Finally, at 29 dpt, both the CON and D-SFB birds were colonized with SFB in the medial and distal ileum (Fig. 3D). Specifically, 0% (0/6) of CON birds and 33% (2/6) of D-SFB birds showed SFB colonization of the proximal ileum at 29 dpt. Complimentary to PCR data, Gram stain microscopy demonstrated distinctive SFB morphologies in each ileum section (Fig. 4A through D).

**Fig 5:**
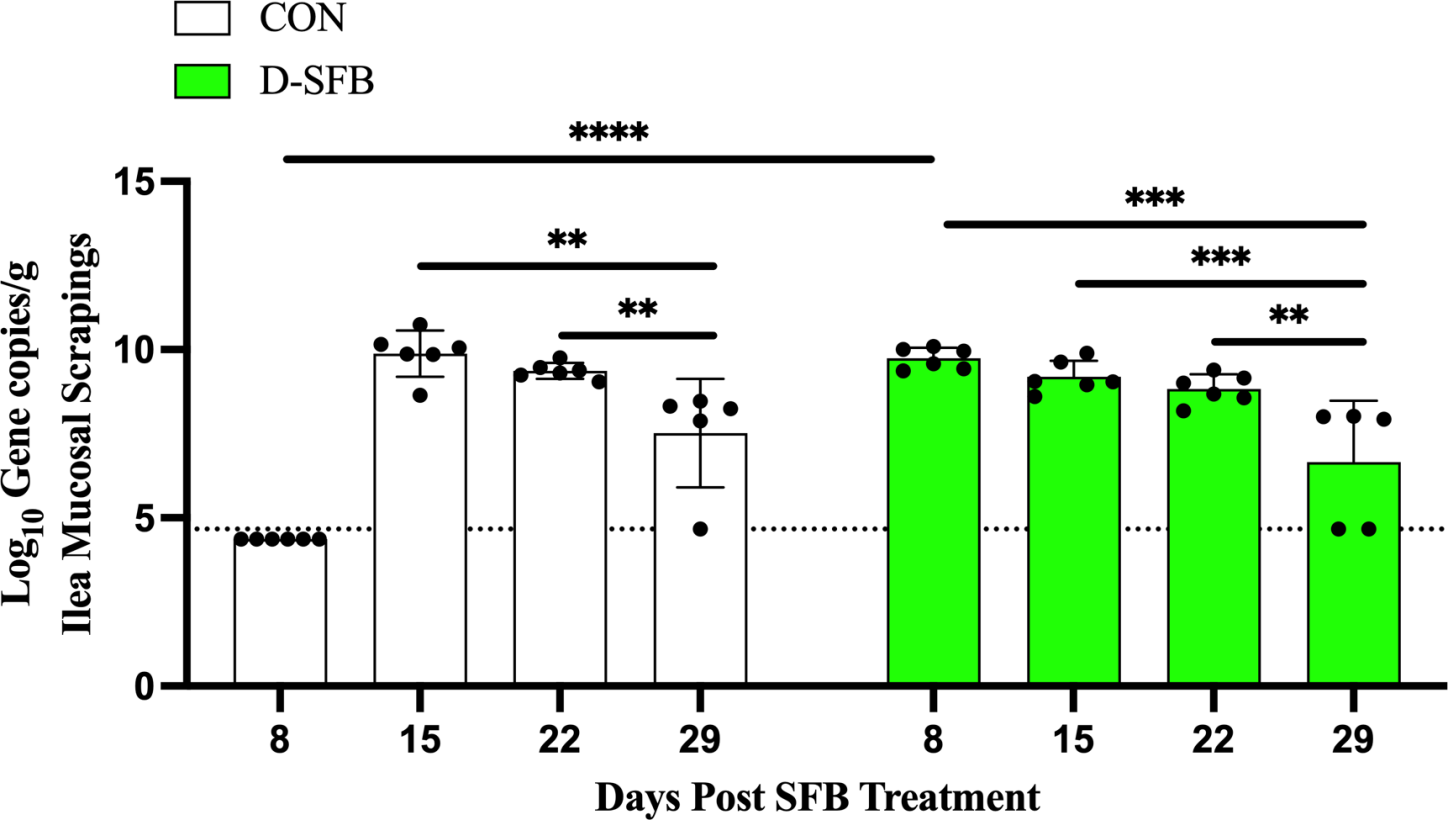
Quantification of ileum-associated SFB in Ross308 broilers. Ileum-associated SFB were quantified from distal ileum scrapings at 8, 15, 22, and 29 days post-treatment (dpt) and compared between control (CON) and SFB-treated (D-SFB) broilers, and comparisons within respective groups are shown between timepoints after initial detection. The limit of detection (LOD) is denoted by a dotted line. Dots represent individual birds. Bars denote the mean with standard deviation. **, *P* < 0.01; ***, *P* < 0.001; ****, *P* < 0.0001.

Next, to quantify the level of SFB, we used SFB-specific quantitative PCR (qPCR) and targeted the SFB primary site of colonization, the distal ileum (22). At 8 dpt, a significantly higher amount of SFB was detected in D-SFB birds compared to CON broilers (*P* < 0.0001; Fig. 5). At 15 dpt, all birds had their ileum colonized with SFB, and there were no significant differences between groups (Fig. 5). The amount of ileal-associated SFB significantly decreased at 29 dpt by approximately 2 log_10_ gene copies/g in CON birds compared to all other times after initial colonization (15 and 22 dpt, *P* < 0.01; Fig. 5). Similarly, in the D-SFB group, the same trend was observed when comparing the level of SFB at 29 dpt to all other times tested (8 dpt, *P* < 0.0001; 15 dpt, *P* < 0.001; and 22 dpt, *P* < 0.01; Fig. 5). Red arrows indicate SFB-like bacteria.

### SFB-based treatment induced transcriptional changes in intestinal barrier function proteins and cytokine genes

**Fig 6:**
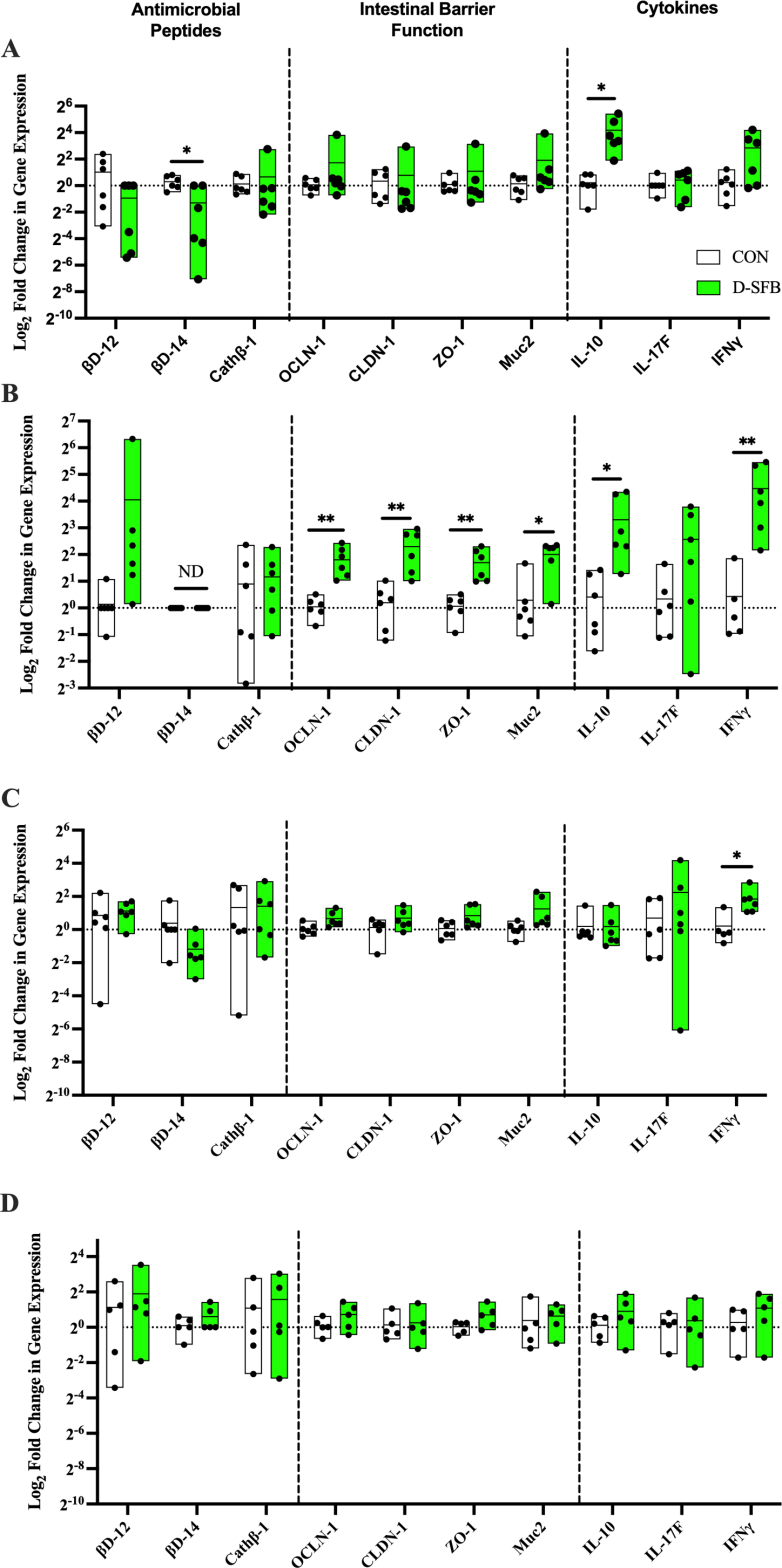
RT-qPCR analysis for broiler ileal immune gene activation. Mucosal scrapings from the distal ileum of control (CON) and SFB-treated (D-SFB) were subjected to RT-qPCR for antimicrobial peptides, intestinal barrier function genes, and SFB-related cytokines. Mucosal scrapings were collected at (**A**) 8, (**B**) 15, (**C**) 22, and (**D**) 29 days post-treatment (dpt). Differences in gene expression were evaluated via the 2^−ΔΔCT^ method. ND, not detected. Dots represent individual birds. *, *P* < 0.05; **, *P* < 0.01.

Log2 fold changes in gene expression of host intestinal function proteins, e.g., AMPs, barrier function proteins, and cytokines, were measured via reverse transcription (RT)-qPCR in D-SFB and CON groups. All genes tested were expressed in CON birds at any time tested except β-defensin 14 at 15 dpt (Fig. 6B). Compared to the CON group, D-SFB birds had higher gene expression of regulatory cytokine IL-10 at 8 and 15 dpt (*P* < 0.05; Fig. 6A and B). At 15 dpt, D-SFB birds had a significantly higher level of gene expression of occludin-1, claudin-1, ZO-1 (*P* < 0.01), and Muc2 (*P* < 0.05) (Fig. 6B). At 15 and 22 dpt, the expression of the pro-inflammatory cytokine IFNγ was significantly higher in the D-SFB group (*P* < 0.01 and *P* < 0.05, respectively; Fig. 6B and C). Finally, β-defensin 14 expression was significantly lower in D-SFB birds compared to CON birds at 8 dpt (*P* < 0.05; Fig. 6A). No significant differences in AMPs nor IL-17F expression were detected at subsequent times between groups.

### SFB-based treatment resulted in changes in the gut microbiota

**Fig 7:**
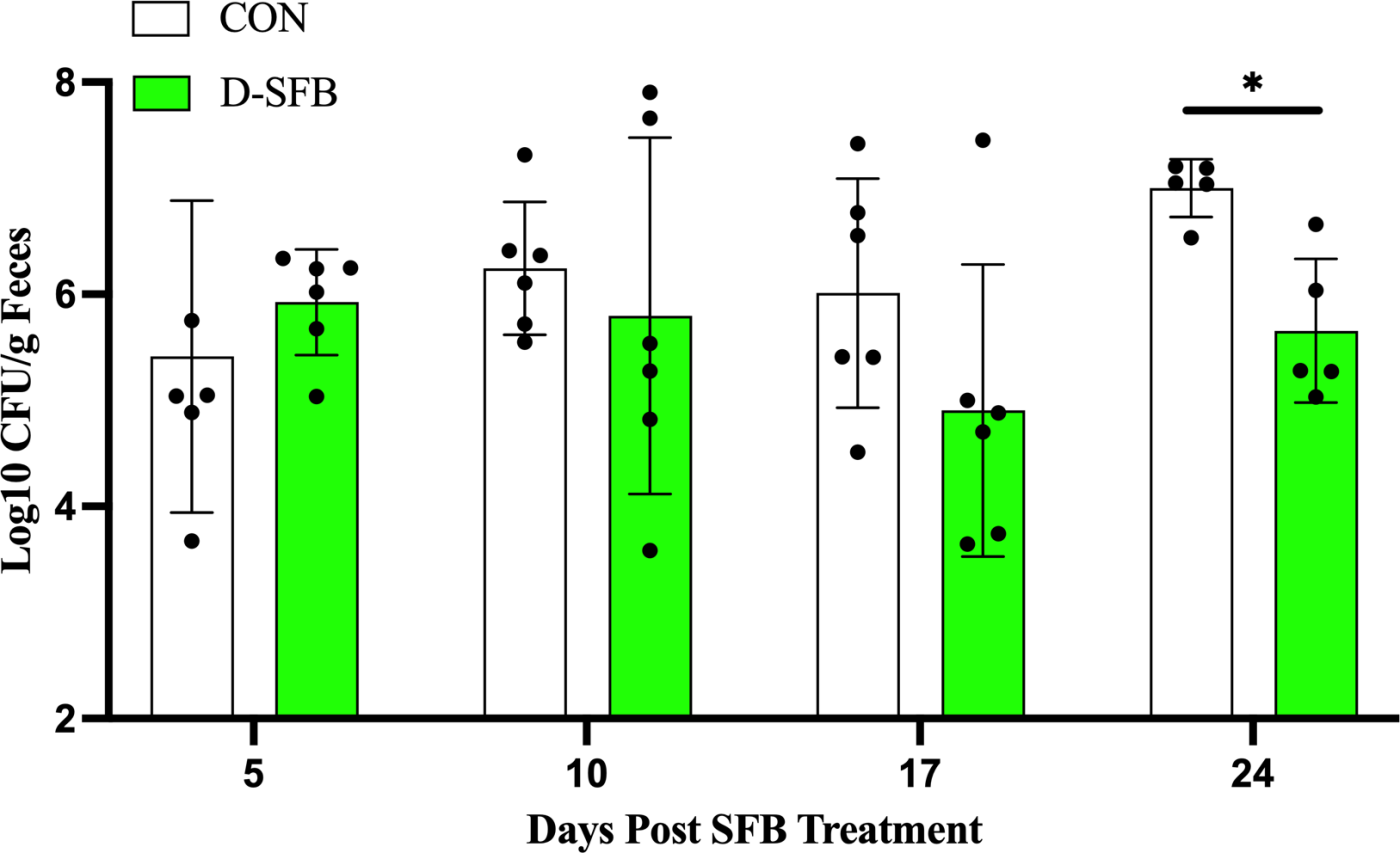
*Enterobacteriaceae* enumeration in feces of Ross308 broilers. Total fecal *Enterobacteriaceae* in control (CON) and SFB-treated (D-SFB) birds were enumerated at 5, 10, 17, and 24 days post-treatment (dpt). Dots represent individual birds. Bars represent the mean with standard deviation. *, *P* < 0.05.

The potential effect of D-SFB treatment on the gut microbiota was evaluated via culture-based methods and genotypically. The level of total *Enterobacteriaceae,* which are responsible for gut inflammation and dysbiosis in chickens (23), was estimated in the feces of the CON and D-SFB birds by colony enumeration on MacConkey agar plates. Overall, in the CON group, the level of *Enterobacteriaceae* numerically increased over time, while the opposite trend was observed in D-SFB-treated birds (Fig. 7). At 24 dpt, the D-SFB group had a 1.5 log_10_ CFU/g lower level of *Enterobacteriaceae* compared to CON birds (*P* < 0.05; Fig. 7). No significant differences in the level of fecal *Enterobacteriaceae* were observed between groups at any other times tested. However, taxon typing qPCR to distinguish changes in the major gastrointestinal tract phyla Firmicutes and Bacteroidetes and the potential beneficial genus *Lactobacillus* spp. showed no significant differences in the proportion of Firmicutes and Bacteroidetes, the ratio Firmicutes/Bacteroidetes, nor the relative abundance of *Lactobacillus* spp. at any times tested in the distal ileum of D-SFB birds compared to CON birds (Fig. S1).

### SFB-based treatment increased intestinal and extraintestinal killing of pathogens

**Fig 8:**
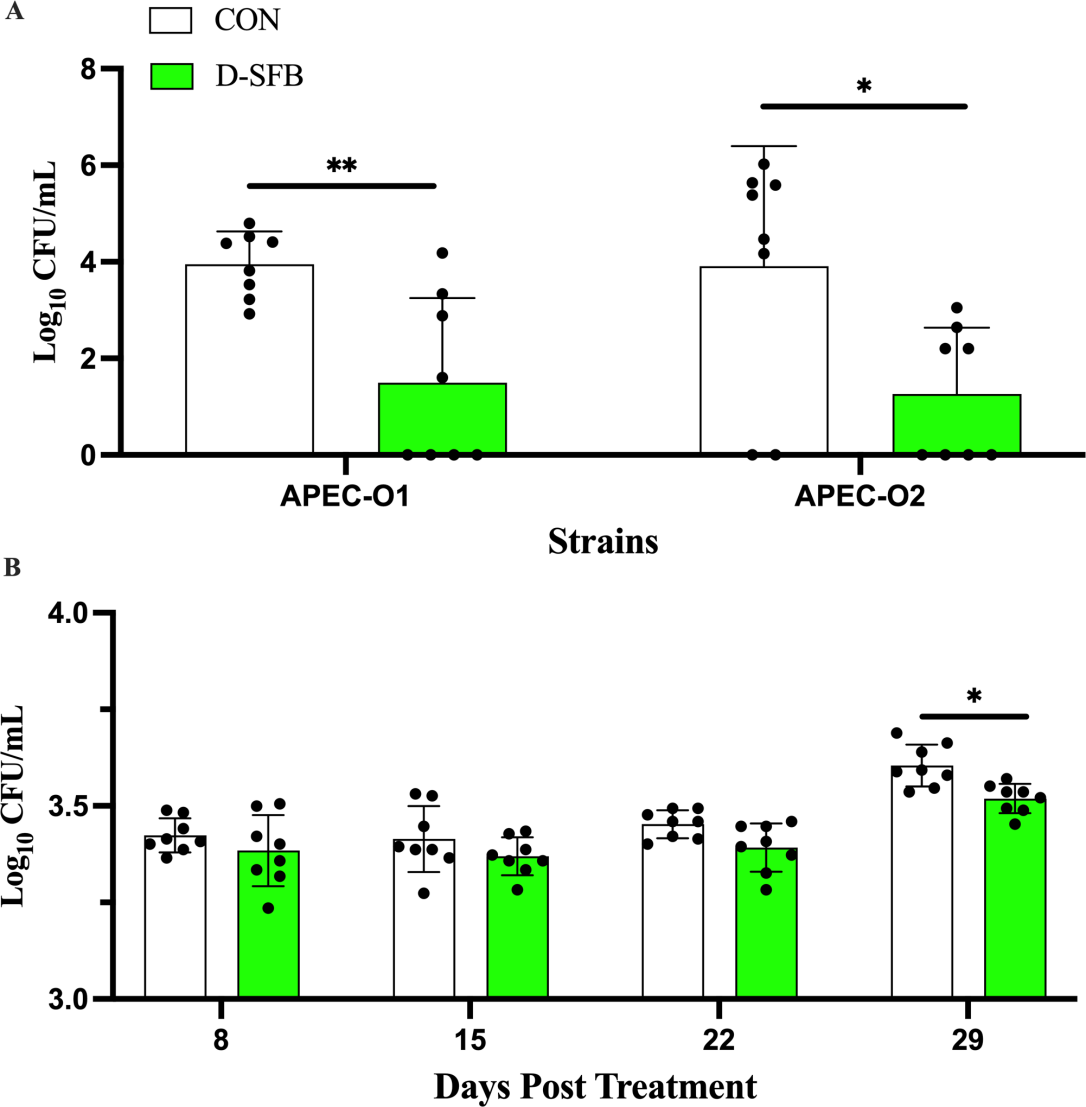
Treatment effect on antibacterial activity of broiler ilea and sera. (**A**) Antibacterial ability of sera to avian pathogenic *E. coli* (APEC). Bacterial loads (log_10_ CFU/mL) of representative APEC-O1 and APEC-O2 strains incubated 6 hours at 40°C with pooled sera from non-treated (CON) and treated (D-SFB) groups at 29 days post-treatment (dpt). Subsequently, assays were serially diluted and plated on violet-red bile agar (VRBA) and incubated at aerobic conditions at 37°C overnight. (**B**) Ileal bactericidal ability to *Campylobacter jejuni*. Representative *Campylobacter jejuni* (CDC Antibiotic Resistance Bank strain #0421) strain was incubated with pooled small intestinal scrapings from CON and D-SFB groups at 8, 15, 22, and 29 dpt for 6 hours at 40°C in microaerophilic conditions. Dots represent individual replicates. Bars represent means with standard deviation. *, *P* < 0.05; **, *P* < 0.01.

The ability of the D-SFB treatment to increase host resistance to bacterial pathogens relevant to poultry (24) was tested *in vitro* using blood serum against avian pathogenic *Escherichia coli* (APEC) that cause extraintestinal infections and colibacillosis in chickens (25) and ileal mucosal scrapings for the clinically relevant *Campylobacter jejuni* that naturally colonize the chicken ileum (26). Blood serum, which plays a key role in preventing further systemic APEC infection during avian colibacillosis (27), was used to investigate their increased bactericidal effects against relevant APEC strains. In the serum bactericidal assay, D-SFB blood serum had a significantly superior killing ability to APEC-O1 and APEC-O2 compared to CON birds (*P* < 0.01 and *P* < 0.05, respectively; Fig. 8A). D-SFB mucosal ileal scrapings had a significantly higher killing ability to *Campylobacter jejuni* than CON birds at 29 dpt (*P* < 0.05; Fig. 8B). There were no significant differences in *Campylobacter jejuni* killing at any other times tested.

## DISCUSSION

Inheriting a healthy microbiota at hatch is crucial for a proper immune development in chickens (28); thus, introducing “maternal” microbiota or commercially available probiotics to chicks at hatch is essential to impart protection in early life and drive immunological changes that benefit the immune system throughout the entirety of life (29). Host-specific SFB are one of the key bacteria inherited from the progenitors after birth (30). As mainly shown in mice, a hallmark of SFB is their ability to intimately bind to the gut epithelium and trigger innate and adaptive immune responses in their respective hosts only (8, 12, 31). Similar to our previous studies on layer chickens (5, 6), this study detected SFB in broiler chickens and showed variabilities in colonization and immune maturation between individuals when not receiving SFB at hatch, indicating the importance of giving these bacteria to newly hatched chicks in industrial settings, where chicks are hatched in the absence of their progenitors.

SFB preferably colonize the distal ileum of mice (8, 10) and broiler chickens (4). Thus, we previously focused on this ileum section to test SFB colonization in layers (5, 6). However, broilers raised for meat production have enlarged ileum to meet the demand for increased nutrient absorption for a faster growth rate (32, 33). Thus, this study investigated SFB colonization in the different sections of the broiler ileum, e.g., proximal, medial, and distal. In a study that tested farm-raised Hubbard broilers, only 20% of birds had their distal ileum colonized with SFB at 1 week post-hatch (4). Here, in Ross308 broilers, our results align with those of layers (5, 6) on the ability of the SFB treatment to increase SFB ileum colonization in a shorter time. Still, uniquely, this study showed that this colonization was not sequestered explicitly to the distal ileum but included the proximal and medial ileum as well. These data warrant future studies on the link between SFB colonization patterns in the specific ileum sections and immune activation.

The colonization of SFB in their respective hosts and the progression of SFB through their lifecycle are directly related to their impact on host immune maturation (11). The SFB lifecycle is a unique process only observed in limited rodent studies (30, 34, 35). SFB spores acquired from the environment travel through the gastrointestinal tract of the host organism, where they germinate in the distal ileum to form small holdfast cells that intimately bind to the epithelium of the intestine (8, 11). Once bound, the SFB holdfast cells will propagate into differentiating filaments that, in turn, release intracellular offspring that directly bind to the host or travel through the large intestine to exit as spores or, in some cases, bind to areas like the ceca (13). In this study, the universal SFB primers used would not have distinguished chicken SFB from those of other sources (humans, mice, etc.) (13). However, knowing SFB mature to filamentous form only in their specific host (36), testing the presence of segmented filamentous morphologies of SFB in chicks in complement to the PCR provides strong evidence for the avian source of the SFB spores in broilers tested. The results of this study clearly show how treatment of broilers with SFB spores accelerates the detection of the filamentous form of SFB in the feces of birds (5 days vs 15 days in non-treated birds), indicating that SFB had already bound and progressed through their lifecycle. The delay in the SFB lifecycle in the non-treated birds could have impaired their gut immune maturation, as only the filamentous form of SFB drives the immune maturation of the gut (34). There remain significant gaps in knowledge of the genetic differences between SFB sourced from different host species; thus, current genome sequencing research is being undertaken to identify strain-specific identifiers.

In this study, although SFB were detected in the feces and cecal content of the non-treated birds at the earliest times (days 5 and 8 post-SFB treatment), this detection was not consistent compared to that of the SFB-treated birds. Furthermore, the sporadic detection of filamentous SFB in the feces of non-treated birds and their absence in the cecal content demonstrates that the SFB present in this group were less advanced in their lifecycle than those of the SFB-treated birds. Owing to their low level, we speculate that the level of SFB present in the non-treated birds was below the limit of detection utilized in our qPCR assays for the distal ileum. Still, as these bacteria grew in the bird's gut and spread through the environment to other birds, the level of SFB increased, thus becoming observable above our limit of detection in qPCR assays at the following times (days 15, 22, and 29 post-SFB treatment).

Yet, it is unclear where SFB in the non-treated birds come from. To prevent the risk of cross contamination of SFB when treated and non-treated birds are housed in the same room (4, 5), in this study, we housed the two groups in separate rooms. However, knowing SFB spores can persist in the environment and readily colonize respective species upon ingestion (6), and the vertical transmission of SFB in mammalian hosts has been well studied (30), we postulate that SFB in the non-treated birds may have been inherited while eggs were developing inside the hens' reproductive tract, as previously shown with pathogenic bacteria like *Salmonella* (37) and early microbiota (38). Alternatively, spores on the eggshells could have resisted cleaning treatments and transferred to the chicks at hatch. Future studies are needed to verify the SFB vertical transmission in chickens.

Upon attachment to the ileum in early life, SFB maintain a symbiotic relationship with their specific host, with host cells providing key nutrients to trigger filamentation of SFB, which trigger gut immune maturation. As SFB mature through their lifecycle, they are limited in expansion due to specific secretory IgA and neutrophil recruitment by the host (39). In fact, SFB have been shown to have a peak of colonization post-introduction that recedes to a small resident population as the host matures. Here, in broilers, the peak of SFB colonization of the ileum in the treated birds occurred approximately 2 weeks post-hatch and dwindled at 4 weeks, similar to what was observed in commercial turkeys (40) and broilers (4). The extended tracking of SFB colonization past 2 weeks in broilers demonstrated a decrease in levels of SFB in both SFB-treated and untreated birds at 29 days post-SFB treatment. This early SFB colonization and its subsequent reduction over time is imperative for the SFB to regulate host immune maturation without causing host pathology (41). Our prior study in layer chickens further demonstrated the binding of SFB to the host epithelium without inducing inflammation (5). Here, in the untreated birds, the delayed SFB ileal colonization (15 days) was outside the timeframe in which SFB drive immune maturation. These findings highlight the importance of providing birds with a microbiota, like our SFB-enriched treatment at hatch, to efficiently activate the gut immune system without the risk of chronic inflammation (42). However, further studies are required to address the gap in knowledge concerning the implementation of SFB treatment in later life and the dosage response to SFB colonization.

Next, we assessed the ileal gut immune activation by SFB in broilers by testing key immune factors related to homeostasis, antibacterial activity, and intestinal barrier function. In mice, SFB specifically induce T_H_17 cells upon attachment to the ileum (42–44) as evidenced by IL-17 production (10, 12). Utilizing the chicken-specific Kinome peptide array, we previously demonstrated an increased T_H_17 cell differentiation and signaling in SFB-treated layer chickens at 7 days post-SFB treatment (5). Therefore, the results presented in this study align with those in our previous work in layer chickens, where the increase in IL-17 production is absent in chickens (6). To gain a clearer picture of the homeostatic impact of SFB colonization, we tested the anti-inflammatory cytokine IL-10 and found that this cytokine was significantly more expressed in SFB-treated broilers than in the control birds at both 8 and 15 days post-treatment. These results directly reflect what was previously demonstrated in layers with increased expression of IL-10 at approximately 2 weeks post-SFB treatment (6). Future studies will include other markers of T_H_17, such as IL-22, to fully encapsulate the dynamics of the influence of SFB on T_H_17 cell-specific differentiation in chickens to compare to what is known in murine models. In mice, SFB-specific T_H_17 cells in the lamina propria interact with other CD4+ T cells to modulate the production of inflammatory cytokines and differentiation, which illustrates the ability of SFB to trigger a homeostatic and unique immunomodulating T_H_17 cell response marked by the production of IL-10 rather than a pathogenic autoimmune response (45). Our previous results in layers thoroughly demonstrated that the immunomodulation caused by early colonization of SFB did not result in inflammation (5). Through the demonstrated production of IL-10 in our current study, we provide strong evidence that supports the non-inflammatory immune activation of SFB when provided in early life.

Furthermore, we assessed the levels of IFNγ, a cytokine that triggers antimicrobial activities in the small intestine (46), and host-derived AMPs like cathelicidins and defensins (45, 47, 48). A significant increase in IFNγ but not AMPs was observed at 15 and 22 days post-SFB treatment. Notably, the production of IFNγ and IL-10 in concert was demonstrated consistently to modulate host responses without causing inflammation (46). This study focused on three AMPs, e.g., cathelicidin β-1 and β-defensins 12 and 14, based on our previous findings in layer chickens that demonstrated increased expression of β-defensin 14 at 2 weeks post-treatment and their importance for maintaining healthy gut microbiota (6, 49–51). Future studies will encompass broader screening techniques to fully understand the AMP production influenced by SFB. However, the findings presented in our current study highlight one of the critical dynamics of crosstalk between the host and SFB. Specifically, neutrophil depletion *in vivo* shows an impaired expression of IL-22 and AMPs, leading to uncontrolled expansion of SFB (39). Further, in mice deficient in α-defensin production, SFB colonize at a higher rate than their wild-type counterparts (52). However, these dynamics are unclear in chickens. Our current study demonstrated significantly lower expression of β-defensin 14 at 8 days post-SFB treatment that correlates to the peak of SFB colonization. At further times tested (15, 22, and 29 days post-SFB treatment), there was no difference in the expression of any AMPs between SFB-treated and the control broilers, but the levels of SFB decreased in both groups at the same rate. These data suggest that this decrease in β-defensin 14 expression could have favored the early SFB colonization of the ileum and their reduction over time (53, 54).

The production of lamina propria IL-10 has been directly correlated with the management of tight junction proteins, mucin production, and activity of Paneth cells (55–58), which are critical components of intestinal health and a barrier between the microbiota and epithelium (59, 60). In layers, ileal SFB colonization decreased gut leakage at 2 weeks post-treatment (5). Here, a higher expression of genes conferring tight junction proteins, such as occludin (OCLD-1), claudin (CLDN-1), and zonula occludens (ZO-1), was observed in SFB-treated broilers at 15 days post-SFB treatment, which could prevent pathogen translocation and improve overall gut health (55, 56). In addition, there was an increase in mucin-2 (Muc2) expression in SFB-treated broilers simultaneously. The Muc2 expression leads to mucus production in goblet cells, which regulate microbiome homeostasis and prevent diseases (57–59). Combining these findings with data from the significantly greater colonization of SFB in the distal ileum at 8 days post-SFB treatment, it is illustrated that the early colonization of SFB is necessary to trigger improvements in intestinal barrier function. This is further illustrated by the fact that non-treated birds demonstrated comparable levels of SFB at 15 days post-SFB treatment and did not display an increase in genes associated with intestinal barrier function or anti-inflammatory cytokine production. Altogether, these data illustrate how an early chicken SFB colonization can trigger gut homeostasis by expressing IL-10 and genes involved in intestinal barrier function.

We tested whether the broiler's ileal SFB-associated colonization and immunity provide resistance to pathogenic bacteria. SFB trigger protection in the ileum against pathogens, such as *Listeria monocitogenes*, *E. coli*, *Salmonella,* and *Citrobacter rodentium* in mice (61–64) and broad resistance to *Salmonella* in layer chickens, as demonstrated by our studies (5, 6). In broiler production, managing bacteria that naturally colonize the ileum, like *Campylobacter jejuni* is essential for animal and consumer health (65). The *in vitro* antibacterial activity assay of ileal scrapings against this bacterium in this study showed the ability of SFB treatment to increase the killing ability against *Campylobacter jejuni*. The occurrence of this activity at around 4 weeks post-treatment indicates the importance of completing the SFB lifecycle to modulate microbial responses. Future studies will attempt to study the impact of SFB on *Campylobacter in vivo*.

To evaluate the impact of the treatment on other commensal bacteria in the gut, we tested the changes in targeted gut bacteria, using culture-based and genotypic assays, including *Enterobacteriaceae,* a representative family of the phylum Pseudomonadota (previously Proteobacteria) and the phylum Firmicutes, which are some of the predominant early colonizers of the chicken ileum (66). The culture-based testing of the fecal *Enterobacteriaceae* determined the ability of SFB-based treatment to reduce *Enterobacteriaceae* in the gut, which could lead to a reduced risk of inflammation and dysbiosis in broiler chickens (67). However, taxon typing qPCR detected no significant effect in Firmicutes, Bacteroidetes, nor probable beneficial bacteria *Lactobacillus spp*. of the ileum. The ratio of Firmicutes to Bacteroidetes serves as an important marker for intestinal health, and alterations in this ratio significantly impact fat deposition and growth rate in broilers (68, 69). Additionally, Lactobacilliaceae, which are a constitutive part of the ileum microbiota, confer positive benefits due to their probiotic potential (70). However, since we detected a decrease in total *Enterobacteriaceae*, changes at genus/species levels of the Firmicutes/Bacteroides phyla could not have been detected due to the resolution of the genotypic method we used. Next-generation sequencing studies are ongoing to adequately address the gaps in knowledge regarding the impact of SFB colonization on the microbial community.

In mice, SFB trigger broad immunological effects in distal organs not directly associated with the intestine (71), such as in the brain, liver, pancreas, joints, and lungs of mice (72–74). A link between SFB ileal colonization and protection from acute lung infections with methicillin-resistant *Staphylococcus aureus* (MRSA) was demonstrated in mice (74). In broiler chickens, APEC is a serious concern for animal health and productivity. APEC cause extraintestinal colibacillosis infections, mainly through the lungs (75–77), and the blood serum is an essential barrier for the protection against systemic infection (27). Our previous work showed that oral prophylactics, such as probiotic and vaccine treatments in layer chickens, impact the serum-killing ability of multiple APEC strains *in vitro* (78). In this study, the increased serum-killing ability of APEC serotypes O1 and O2 in SFB-treated broilers indicates a potential for broad extraintestinal protection of the SFB treatment against bacteria in chickens. The ability of SFB to interact with the host and drive immune maturation through specific microbe-immune dynamics likely creates an environment wherein the *Enterobacteriaceae* population is better controlled. The interaction during the early colonization of SFB and the host, as well as the increase in serum bactericidal activity to APEC, warrants follow-up studies to better understand the full extraintestinal impact of SFB in chickens.

### Conclusions

**Fig 9:**
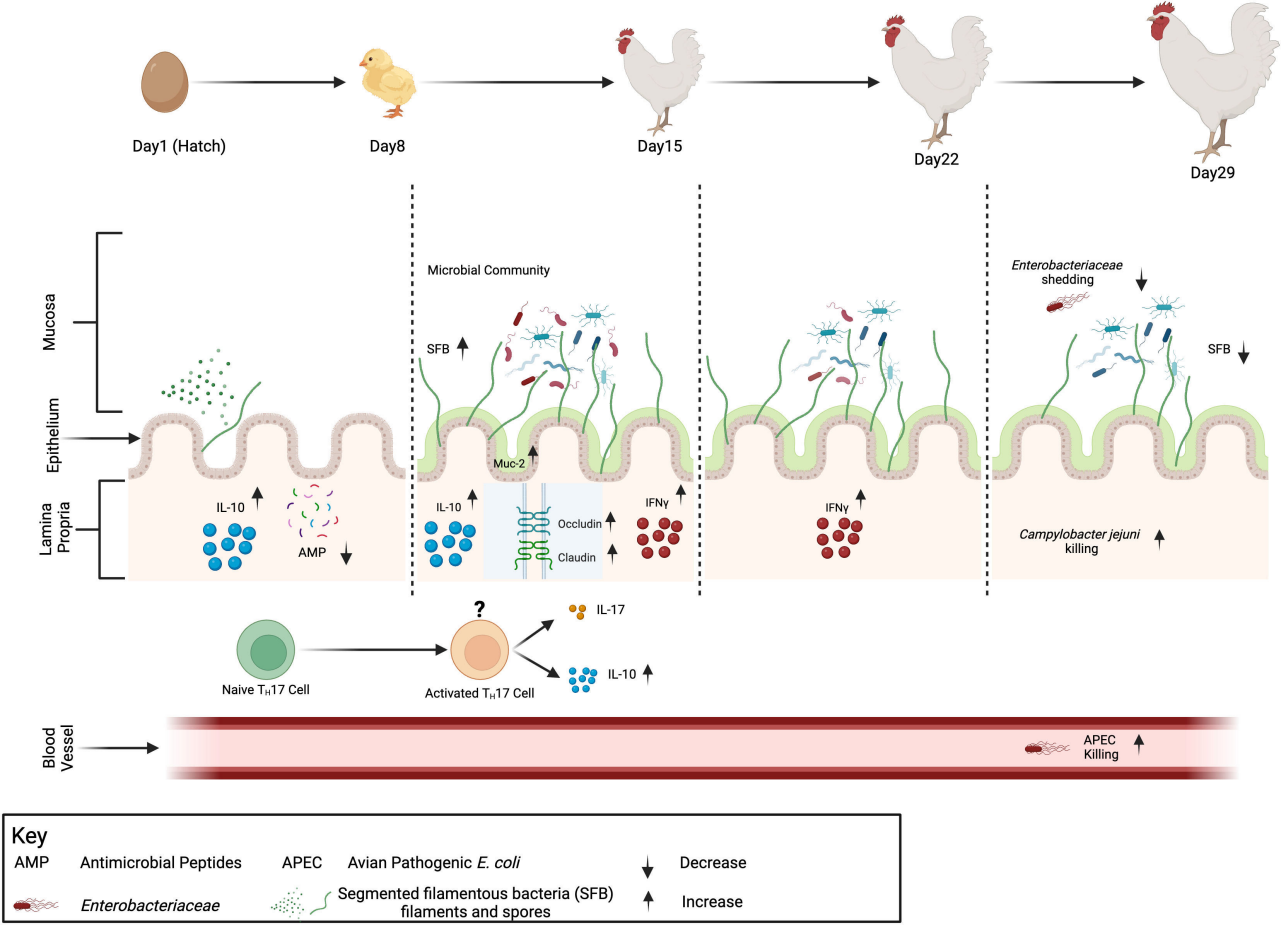
Summary of procedures and results of this study. Day-of-hatch Ross308 broilers were orally inoculated with PBS (CON) or Dekalb-derived segmented filamentous bacteria (SFB) (D-SFB; pictured above). The level of SFB in the distal ileum, as measured via qPCR, peaked at 2 weeks and then decreased. Measuring different factors in gut immune maturity via RT-qPCR at different times shows an increase of key cytokines and tight junction proteins but not antimicrobial peptides (AMPs). The impact of the treatment on the intestinal microbiota, as measured through taxon typing qPCR and screening of feces for *Enterobacteriaceae,* shows a decrease in *Enterobacteriaceae* at week 4. Finally, specific *in vitro* killing assays demonstrated increased killing against clinically and industrially relevant pathogens like *Campylobacter jejuni* and avian pathogenic *E. coli* (APEC). This figure was created with Biorender.com.

This study demonstrates the ability of an SFB-enriched inoculum to ensure ileal SFB colonization in broilers in early life and minimize variation in SFB colonization among individuals. The colonization of SFB in broiler chickens is not limited to the distal ileum but to the entire length of the ileum. Furthermore, the colonization of SFB in early life leads to enhanced intestinal barrier function and homeostasis in broilers. Interestingly, in broilers, there was no increase in IL-17 production indicative of T_H_17 cell activation like previously shown in mice. However, with the increase in both IL-10 and IFNγ, we postulate that the SFB treatment resulted in the activation of T_H_17 cells, leading to a phenotypic shift to IL-10^+^ T_H_17 cells. This SFB immune activation triggered broad bacterial killing activity on both intestinal and extraintestinal pathogens *in vitro* (Fig. 9). Finally, as demonstrated, layer-derived SFB can cross-colonize in broiler chickens; this eliminates the need for multiple SFB-based products to be derived from both layers and broilers. Additional studies are underway utilizing the complete genome sequence of chicken derived SFB to further study specific host-microbe interactions with SFB-specific genes.

## MATERIALS AND METHODS

### SFB inoculum preparation

Methods for the enrichment of ileal spores were described previously (6, 49, 79) with some modifications to improve the purity of inoculum. Briefly, the medial ileum (approximately 4 cm proximal of the ileum-cecum junction) of 2-week-old commercial Dekalb layers (*n* = 20) were removed and flushed twice with sterile phosphate-buffered saline (PBS). The ilea were then opened longitudinally and scraped with an ethanol-sterilized razor along the epithelial surface. Scrapings were pelleted at 10,000 × g for 10 minutes, resuspended in 1% peptone and 15% glycerol solution, and stored at −80°C for future experimentation. Individual aliquots from Dekalb scrapings were thawed at room temperature (RT) and subsequently treated with chloroform (3% of total solution, vol/vol) for 30 minutes at 37°C as previously described (6). Suspensions were screened on MacConkey agar (Thermo Fisher, Waltham, MA, USA) and blood agar (Thermo Fisher) to ensure the absence of culturable microbes at aerobic, anaerobic, and microaerophilic conditions. Suspensions that indicated no colonies after 2 days of culture were pooled, resuspended in peptone-glycerol solution, and stored at −80°C until use in animal experiments. In our previous work, this method has been shown to eliminate all readily culturable spore-forming microbes (5, 6), and 16S rRNA sequencing demonstrated the specific enrichment of SFB in the inoculum; however, a passage in germ-free chickens, as previously done in mice (80), may be necessary to claim a pure SFB inoculum; thus SFB-enriched inoculum is used in this study. The inoculum will now be referred to as D-SFB for Dekalb-derived SFB. The presence and the concentration of SFB in the inoculum were determined as described in the sections below.

### Broiler treatment and sampling

Day-old Ross308 broilers were massed and evenly distributed into two pens (*n* = 23/pen) in two separate rooms. Immediately after placement, birds were orally inoculated with 50 µL sodium bicarbonate and either 100 µL (~10^4^ SFB) D-SFB or 100 µL PBS (CON). Food and water were provided 30 minutes after treatment, and animals had *ad libitum* access until necropsy. Staff were required to enter the room containing the CON group first. Staff wore medical scrubs, lab coats, hair nets, polyvinyl boot covers, gloves, surgical masks, and eye protection. Before entering the D-SFB room, staff were required to remove all personal protective equipment (PPE) and change to fresh, clean PPE. Feces were collected at 5, 10, 17, and 24 dpt. At 8, 15, 22, and 29 dpt, birds (*n* = 5–6/group) were massed and then euthanized via CO2 asphyxiation. Blood sera were freshly collected as previously described (81). Ileal scrapings were collected aligning to the proximal ileum (immediately distal to the Meckel's diverticulum), medial, and distal (2 cm proximal to the ileum-cecum junction) (32, 82). Cecal contents were collected and snap-frozen in liquid nitrogen. All samples were stored at −80°C for future use.

### Ileum-associated SFB quantification via qPCR

DNA was extracted from the D-SFB inoculum, ileal tissue sections, cecal content, and feces via boiling lysis as described (83) with minor modifications. Ileal tissue sections and inoculum were pelleted at 10,000 × g for 10 minutes and washed once with PBS. Approximately 0.1 g of stored feces and cecal content was added to Eppendorf tubes using ethanol sterilized spatula and completed to 1 mL with PBS. Feces and cecal content were homogenized by vortexing, gross material was allowed to settle, and the liquid portion was pelleted and washed with PBS twice as above. All samples were pelleted after washing, resuspended in lysis buffer (84), and boiled at 95°C for 15 minutes. Extracts were treated with a final concentration of 1% sodium dodecyl sulfate (SDS), followed by a subsequent incubation at 95°C for 10 minutes. The DNA was purified through phenol-chloroform washes and ethanol precipitation (84). The DNA quantity of the inoculum was measured using a Qubit 4.0 fluorometer (Thermo Fisher, Waltham, MA, USA). For all other DNA measurements, the DNA quantity and quality were assessed via Nanodrop 2000 (Thermo Fisher). Quantification of SFB was performed in the distal ileum as previously described (6) using a QuantStudio 3.0 thermocycler (Thermo Fisher) and the Maxima SYBR/Green Master Mix (Thermo Fisher). Linear regression was used to determine the limit of detection (LOD) for each reaction (84, 85). The presence or absence of SFB in feces, cecal content, and the proximal and medial ileal scrapings were tracked via conventional PCR utilizing GoTaq Green Master Mix (Promega, Madison, WI, USA) under the same above conditions. The presence of SFB in the distal ileum was determined through amplification in qPCR assays, and PCR gel images of the proximal and medial ileum sections are shown in the supplemental material (Fig. S2). Fecal screenings were only performed at 5 and 10 dpt to show the difference in SFB colonization between birds in the early phase of life and to reduce the stress of fecal collection as weekly necropsies were performed beginning at 8 dpt.

### Visualization of SFB through microscopy

Feces, cecal contents, and scrapings of the ileal sections at all timepoints were tested for SFB-like morphologies via Gram staining (86). Further confirmation of SFB-like morphologies was performed with cecal content using fluorescent *in situ* hybridization (FISH) with SFB-specific probes, as previously described (6, 87, 88). Brightfield and fluorescence microscopy were performed utilizing the Keyence BZ-X800 microscope (Keyence, Osaka, Osaka, Japan). Fluorescent microscopy images were overlaid with phase contrast microscopy to demonstrate the total bacteria in each sample.

### Gut immune assessment via RT-qPCR

RT-qPCR tested ileal scrapings from treated and non-treated groups for expression of immune genes encoding for host-derived AMPs (β-defensins 12 and 14 and cathelicidin β-1) (49), cytokines (IL-10, IFNγ, and IL-17F) (81, 89), and intestinal barrier function proteins (Muc2, claudin-1, occludin-1, and ZO-1) (60). All primers and probes used in this study can be found in Table 1. Total RNA was extracted from mucosal scrapings of the distal ileum using TRIzol (Thermo Fisher, Waltham, MA, USA) according to the manufacturer's instructions. RNA concentration and quality were assessed via Nanodrop 2000 (Thermo Fisher). Reverse transcription assays were performed via the high-capacity cDNA reverse transcription kit (Thermo Fisher), according to the manufacturer's instructions. The RT-qPCR assays were performed using a QuantStudio 3.0 thermocycler. Differences in gene expression were determined via 2^−ΔΔCT^ method using the gene encoding glyceraldehyde 3-phosphate dehydrogenase (GAPDH) as a stably expressed housekeeping control gene (90).

**TABLE 1:** Primers and probes for PCR, qPCR, RT-qPCR, and FISH used in this study

Primer or probe	Sequence	Purpose	Source
SFB 16S rRNA (mouse)	F: 5′-AGGAGGAGTCTGCGGCACATTAGC-3 R: 5′-TCCCCACTGCTGCCTCCCGTAG-3′	qPCR	(13)
SFB 16S rRNA (rat)	GGGTACTTATTGCGTTTGCGACGGCAC	FISH	(4)
Eubacteria	F: 5′-ACTCCTACGGGAGGCAGCAGT R: 5′-ATTACCGCGGCTGCTGGC	Taxon qPCR	(91)
Firmicutes	F: 5′-GGAGYATGTGGTTTAATTCGAAGCA R: 5′-AGCTGACGACAACCATGCAC	Taxon qPCR	(91)
Bacteroidetes	F: 5′-GTTTAATTCGATGATACGCG R: 5′-TTAAGCCGACACCTCACG	Taxon qPCR	(91)
*Lactobacillus* spp.	F: 5′-TGGATGCCTTGGCACTAG R: 5′-AAATCTCCGGATCAAAGCTTAC	Taxon qPCR	(91)
GAPDH	F: 5′-GCACGCCATCACTATCTTCC R: 5′-CATCCACCGTCTTCTGTGTG	RT-qPCR	(90)
β-Defensin 12	F: 5’- AGACAGCTGTAACCACGACA-3 R: 5’- CTGCAGTTCGGACACCTTCA-3	RT-qPCR	(49)
β-Defensin 14	F: 5′-ATGGGCATATTCCTCCTGT-3 R: 5′-CACTTTGCCAGTCCATTGT-3	RT-qPCR	(49)
Cathelicidin β-1	F: 5’- CCGTGTCCATAGAGCAGCAG-3 R: 5’- AGTGCTGGTGACGTTCAGATG-3	RT-qPCR	(90)
IL-10	F: 5′-CATGCTGCTGGGCCTGAA-3 R: 5′-CGTCTCCTTGATCTGCTTGATG-3	RT-qPCR	(81)
IFNγ	F: 5′-ACACTGACAAGTCAAAGCCGC-3 R- 5’- AGTCGTTCATCGGGAGCTTG-3	RT-qPCR	(81)
IL-17F	F: 5’- CTCCGATCCCTTATTCTCCTC-3 R: 5’- AAGCGGTTGTGGTCCTCAT-3	RT-qPCR	(89)
Mucin-2	F: 5’- AAACAACGGCCATGTTTCAT-3 R: 5’- GTGTGACACTGGTGTGCTGA-3	RT-qPCR	(60)
Claudin-1	F: 5’- GGTGAAGAAGATGCGGATGG-3 R: 5’- TCTGGTGTTAACGGGTGTGA-3	RT-qPCR	(60)
Occludin-1	F: 5’- GATGGACAGCATCAACGACC-3 R: 5’- CTTGCTTTGGTAGTCTGGGC-3	RT-qPCR	(60)
ZO-1	F: 5’- GCCAACTGATGCTGAACCAA-3 R: 5’- GGGAGAGACAGGACAGGACT-3	RT-qPCR	(60)

### Enumeration of *Enterobacteriaceae* in feces

At 5, 10, 17, and 24 dpt, feces were aseptically collected from five to six individual birds in each group. Small portions (~0.1 g) of feces were aseptically added to sterile Eppendorf tubes, completed to 1 mL with PBS, and vortexed vigorously to homogenize. After briefly allowing gross material to settle, we serially diluted the fecal suspensions and plated it on MacConkey agar (Thermo Fisher, Waltham, MA, USA). Plates were incubated overnight at 37°C for enumeration.

### Taxon typing of ileum mucosal microbiome

The distal ileum microbiome composition was assessed by taxon typing qPCR (91). DNA was isolated from the distal ileum from four birds per group at each timepoint. Relative abundances were calculated using serial dilutions of target DNA, calculating a linear regression based on the Ct data points using GraphPad Prism software (Version 9.1.1; GraphPad, San Diego, CA), inferring the efficiency from the slope of the line, and utilizing Eubacteria as a reference as previously described (92). DNA from each bird was measured in duplicate at each timepoint. Specifically, the Firmicutes and Bacteroidetes phyla composition and ratio as a marker of intestinal health, as well as the Lactobacillus genus as evidence for the relative abundance of beneficial microbes, were also investigated. All qPCR assays were run in duplicate on a QuantStudio3 thermocycler (Thermo Fisher, Waltham, MA, USA).

### Broiler sera and ileal bactericidal testing

Broiler sera collected at 29 dpt were tested in bactericidal assays against APEC reference strains, APEC-O1 and APEC-O2 (75, 93), as previously described (78). Briefly, blood sera were evenly pooled from five birds per group, added to the bacterial suspension at a ratio of 9:1, and incubated for 6 hours at 40°C to imitate *in vivo* conditions in chickens. Following incubation, assays were serially diluted and plated on Violet Red Bile agar (VRBA) (Thermo Fisher, Waltham, MA, USA) and incubated overnight at 37°C and aerobic atmosphere. Samples were run in quadruplicate, and assays were independently repeated.

Distal ileal scrapings were tested for their killing activity against *Campylobacter jejuni* (CDC Antibiotic Resistance Bank strain #0421). Samples from five birds per group from 8, 15, 22, and 29 dpt were thawed on ice and pelleted at 10,000 × g for 10 minutes. The supernatants were pooled evenly from each group. Importantly, supernatants were plated on Mueller Hinton agar (MHA) to ensure the absence of bacteria. *Campylobacter jejuni* suspensions were prepared from 2-day MHA plate colonies supplemented with tetracycline. Colonies were mixed until an OD_600_ of 0.1 was achieved in PBS and were diluted to a concentration of 10^3^ CFU/50 µL. Bacterial suspensions and pooled ileal scraping supernatant were mixed 1:1 and incubated for 6 hours at 40°C at microaerophilic conditions (2% O_2_ and 10% CO_2_). Mixtures were serially diluted and plated on MHA supplemented with tetracycline and incubated in microaerophilic conditions for 2 days at the above conditions. Pools were run in quadruplicate and independently repeated.

### Statistics

Statistical analyses of the data were performed using the GraFphPad Prism software (Version 9.1.1; GraphPad, San Diego, CA). Differences between D-SFB and CON groups in total *Enterobacteriaceae*, SFB quantification in distal ileal scrapings, *Campylobacter jejuni* killing, taxon typing qPCR, and RT-qPCR data for each gene were measured at each timepoint using multiple *t*-tests with Holm-Sidák's correction for multiple comparisons. Differences in APEC bactericidal assays were determined by Mann-Whitney's test followed by Holm-Sidák’s correction.

## SUPPLEMENTAL MATERIAL

Figure S1 msphere.00492-24-s0001.docx Taxon typing of ileum mucosal microbiome.

Figure S2 msphere.00492-24-s0002.docx SFB colonization throughout the broiler ileum.

ASM does not own the copyrights to Supplemental Material that may be linked to, or accessed through, an article. The authors have granted ASM a non-exclusive, world-wide license to publish the Supplemental Material files. Please contact the corresponding author directly for reuse.
