# Supplementary material for: Layer segmented filamentous bacteria colonize and impact gut health of broiler chickens
Source: mSphere. 2024 Oct 18;9(11):e00492-24. doi: 10.1128/msphere.00492-24 (PMC11580430; doi:10.1128/msphere.00492-24)
Supplement: Figure S1 — Taxon typing of ileum mucosal microbiome. [file msphere.00492-24-s0001.docx]

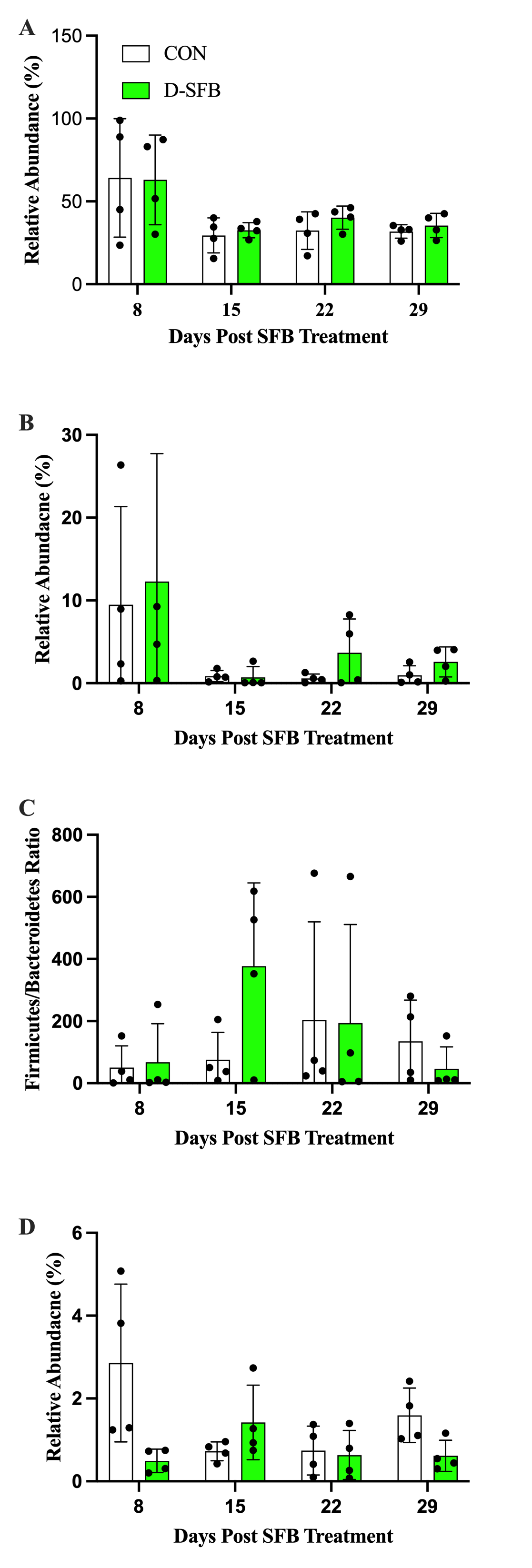


**Supplementary Figure 1.** Taxon typing of ileum mucosal microbiome. The relative abundances of Firmicutes (A), Bacteroidetes (B), the ratio Firmicutes/Bacteroidetes (C), and *Lactobacillus spp*. were quantified from distal ileum scrapings at 8-, 15-, 22-, and 29-days post-treatment (dpt) and compared between control (CON) and SFB-treated broilers (D-SFB). Dots represent individual birds. Bars denote mean with standard deviation.
