# Supplementary material for: Layer segmented filamentous bacteria colonize and impact gut health of broiler chickens
Source: mSphere. 2024 Oct 18;9(11):e00492-24. doi: 10.1128/msphere.00492-24 (PMC11580430; doi:10.1128/msphere.00492-24)
Supplement: Figure S2 — SFB colonization throughout the broiler ileum. [file msphere.00492-24-s0002.docx]

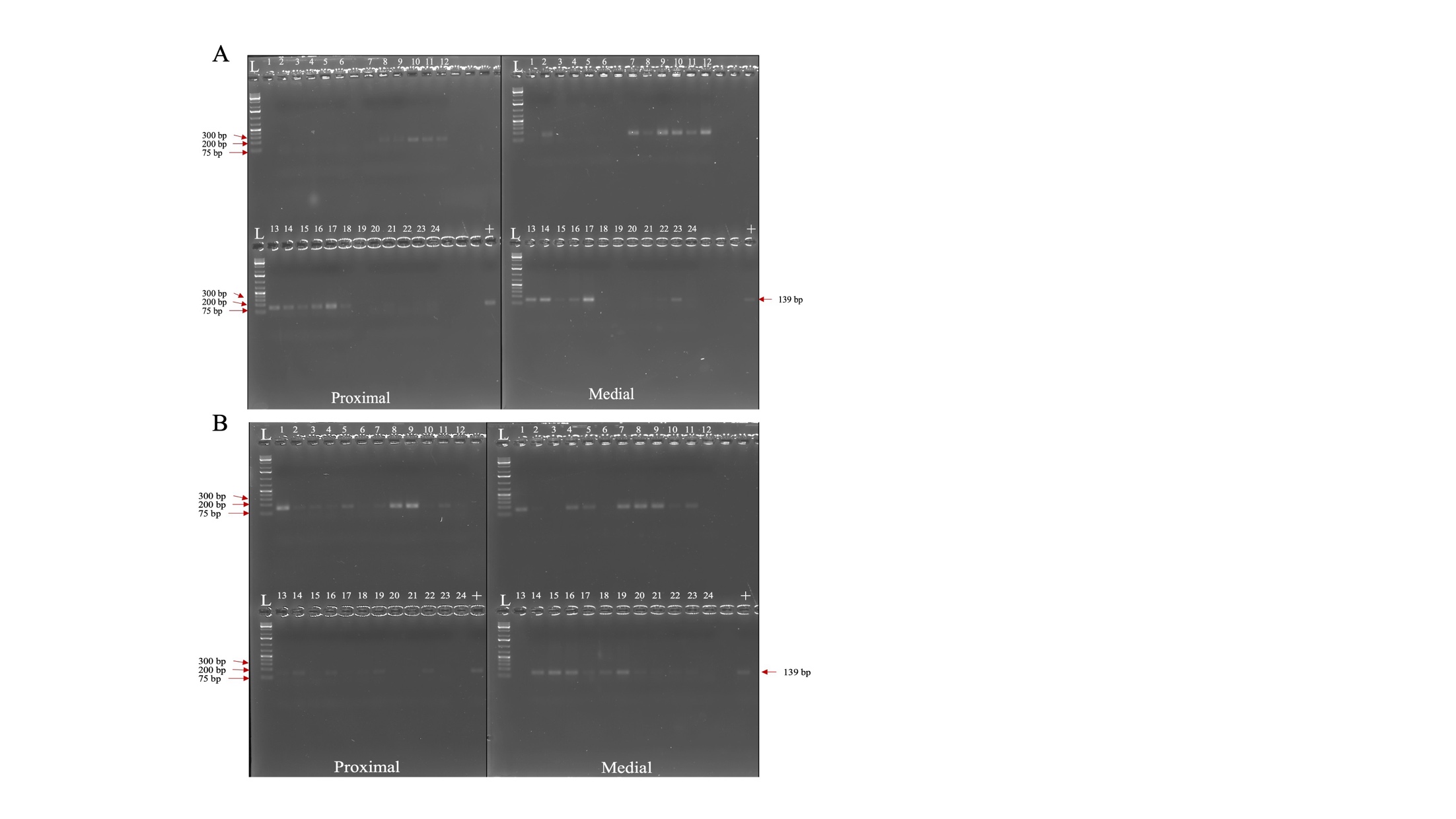


**Supplementary Figure 2**. SFB Colonization throughout the broiler ileum. Representative gel images of the proximal and medial ileum of A) control (CON) and B) SFB-treated (D-SFB) broilers. In each gel image: Lanes 1-6, 8 days post-treatment (dpt); lanes 7-12, 15 dpt; lanes 13-18, 22 dpt; lanes 19-23, 29 dpt; and + denotes positive control (SFB^+^ intestinal scrapings from previous experiment (6)). The Gene Ruler 1kb plus ladder (L) (Thermo Fischer, Waltham Massachusetts, United States) was used for measurement of DNA migration.
